# Elevated methane flux in a tropical peatland post-fire is linked to depth-dependent changes in peat microbiome assembly

**DOI:** 10.1038/s41522-024-00478-9

**Published:** 2024-01-23

**Authors:** Aditya Bandla, Hasan Akhtar, Massimo Lupascu, Rahayu Sukmaria Sukri, Sanjay Swarup

**Affiliations:** 1 NUS Environmental Research Institute, National University of Singapore, Singapore, Singapore; 2 Singapore Centre for Environmental Life Sciences Engineering, National University of Singapore, Singapore, Singapore; 3 Department of Geography, National University of Singapore, Singapore, Singapore; 4 School of Liberal Arts and Sciences, RV University, Bengaluru, Karnataka India; 5 Institute for Biodiversity and Environmental Research, Universiti Brunei Darussalam, Gadong, Brunei Darussalam; 6 Department of Biological Sciences, National University of Singapore, Singapore, Singapore

**Keywords:** Soil microbiology, Microbial ecology

## Abstract

Fires in tropical peatlands extend to depth, transforming them from carbon sinks into methane sources and severely limit forest recovery. Peat microbiomes influence carbon transformations and forest recovery, yet our understanding of microbiome shifts post-fire is currently limited. Our previous study highlighted altered relationships between the peat surface, water table, aboveground vegetation, and methane flux after fire in a tropical peatland. Here, we link these changes to post-fire shifts in peat microbiome composition and assembly processes across depth. We report kingdom-specific and depth-dependent shifts in alpha diversity post-fire, with large differences at deeper depths. Conversely, we found shifts in microbiome composition across all depths. Compositional shifts extended to functional groups involved in methane turnover, with methanogens enriched and methanotrophs depleted at mid and deeper depths. Finally, we show that community shifts at deeper depths result from homogeneous selection associated with post-fire changes in hydrology and aboveground vegetation. Collectively, our findings provide a biological basis for previously reported methane fluxes after fire and offer new insights into depth-dependent shifts in microbiome assembly processes, which ultimately underlie ecosystem function predictability and ecosystem recovery.

## Introduction

Tropical peatlands store 80-105 Gt carbon (approx. 20% of global peatland carbon)^1,2^ and are highly resistant to wildfires in their natural state^3,4^. However, deforestation and drainage-based agriculture, coupled with climate extremes, have rendered them highly susceptible to frequent and severe wildfires^5^. In contrast to wildfire impacts on mineral soils, which are typically limited to the surface, peat fires burn down into the peat, thereby affecting deeper depths^6,7^. Recurrent wildfires strongly alter peat geochemistry^8,9^, hydrology^10^, carbon dynamics^9^, and significantly limit forest recovery^11^. Although carbon dynamics, nutrient cycling, and forest recovery after fire are closely linked to belowground microbiomes, little is known about their responses following the fire event.

Our understanding of the post-fire changes in soil microbiomes is mainly from studies on mineral soils. Fires in these ecosystems reduce microbiome diversity^12–14^, strongly shift composition^13,15,16^, and alter the abundance of key functional groups involved in carbon turnover^13,17–19^. In contrast, our knowledge of post-fire changes in peat microbiomes currently remains limited to changes in microbial abundance. For instance, a drastic reduction in bacterial abundance at the peat surface (approx. 99%) was reported immediately after the fire in a tropical peatland, with little to no change observed three years post-fire. In addition to changes at the surface, a two-third decrease in bacterial abundance was observed in sub-surface peat (30–50 cm), highlighting the effects of peat fires deep below the surface^20^.

In addition to effects on microbiome diversity and composition, fires on mineral soils have been shown to alter the relative balance between deterministic and stochastic assembly processes, which underlies compositional predictability^21^. Previous studies have shown that the shifts in assembly processes post-fire depends on time since fire^19,22,23^. An increase in stochastic processes was observed for a brief period (4–16 weeks) post-fire, likely due to increased dispersal, limited competition, and weak environmental cues^22^. Following this initial period, deterministic processes such as homogeneous selection have been shown to increase 1–4 years post-fire^24^, during which fire-induced changes in soil chemistry and aboveground vegetation act as strong environmental filters on microbiome composition. Severe, recurrent wildfires homogenize environmental conditions and result in convergent or homogeneous selection^13^, while those of low severity lead to divergent or heterogeneous selection due to variable effects on the environment^25^. Finally, recovery from fire on longer time scales (>25 years) is characterised by an increase in stochastic processes as the environmental conditions begin to normalize. Although such studies provide valuable insights into assembly processes at the community level, they do not provide information on processes governing the assembly of individual clades, which may differ in their response to fires. However, recent improvements in null modelling approaches now allow us to estimate the relative importance of ecological processes governing the assembly of individual clades^26,27^.

A tropical peatland that had burned multiple times previously provided us with an opportunity to investigate post-fire effects on carbon dynamics^9^. We had shown that methane emissions remain elevated post-fire and were driven by altered hydrology and peat pore water quality. Carbon isotope signatures showed that methane emissions were most likely linked to the carbon from post-fire vegetation^9^. In a subsequent laboratory study, we confirmed that methane emissions were indeed moderated by temperature, anoxia, microtopography, and methanogenic substrate quality and quantity linked to post-fire vegetation^28^. Here, our objectives were to investigate post-fire shifts in peat microbiome composition, identify microbial groups that potentially underlie methane turnover, and estimate assembly processes that contribute to these post-fire shifts. We accomplished this by profiling microbial communities across depth using marker gene analysis and coupling it with phylogenetic bin-based null modelling to infer assembly processes both at the community and clade levels. Collectively, our findings provide evidence of post-fire shifts in microbiome composition, putative functional groups involved in methane turnover, and assembly processes across peat depth. These results advance our understanding of belowground changes in microbiome composition in an ecosystem where fire impacts occur at deeper depths.

## Results

To date, many studies on post-fire effects on belowground microbiomes have focussed mainly on mineral soils. Yet, in these soils, fire impacts are typically limited to the surface as opposed to peat ecosystems, where such impacts can occur at depth. We address this knowledge gap by profiling microbial communities across three different depths, which capture the altered relationships between the peat surface, water table, and vegetation after fire.

### Peat pore water quality remains strongly altered three years post-fire

We first analysed pore water quality across peat depth as it reflects environmental conditions most relevant to the assembly and functioning of the peat microbiome. Our analysis showed that water temperature (ANOVA, F = 9.4, *p* = 0.006) and pH (ANOVA, F = 10.2, *p* = 0.007) remained significantly elevated three years since fire (Supplementary Figure 1). While there were no significant differences in pH across depths (ANOVA, *p* = 0.16), water temperature was significantly higher (Pairwise *t* test, *p* < 0.001 for all comparisons) at the surface compared to mid and deeper depths. In contrast, there were no significant differences in EC (ANOVA, *p* = 0.07) and DO (ANOVA, *p* = 0.07) levels between burnt and intact peat, nor did they vary across different depths.

### Peat fires shift microbiome composition across depth

**Fig. 1:**
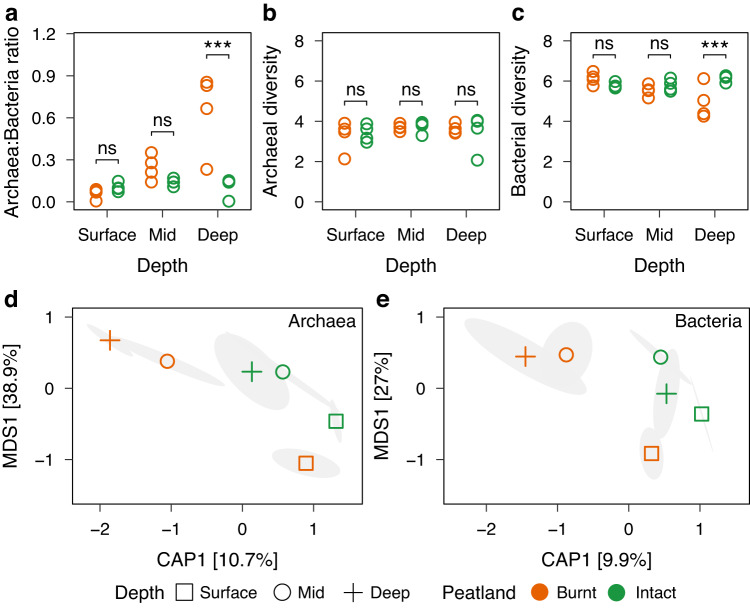
Post-fire shifts in microbiome diversity and composition. **a** Archaea-to-Bacteria ratio increased significantly in deep peat post-fire (Pairwise *t* test, t: 5.88, *p* < 0.001), indicating a shift in relative abundances. **b** No significant difference was observed in archaeal diversity post-fire across any depth. **c** Bacterial diversity significantly decreased in deep peat post-fire (Pairwise *t* test, t: -4.05, *p* = 0.006). **a–c** Show four independent biological replicates per group. Significance is expressed as ***p < 0.001; **p < 0.01; *p < 0.05. Canonical analysis of principle coordinates of surface, mid, and deep peat show shifts in (**d**) archaeal and (**e**) bacterial communities after fire. Symbols in (**d**, **e**) represent community centroids for each depth with 95% confidence ellipses (*n* = 4 for all groups).

We then analysed post-fire changes in peat archaeal-to-bacterial ratios, alpha diversity, and composition using ribosomal small sub-unit marker gene analysis. We detected 3,686 bacterial and 242 archaeal ASVs. Overall, bacteria were relatively more abundant compared to archaea across all samples (mean bacterial relative abundance: 84.39 ± 12.34%). However, archaea increased in relative abundance, and conversely, bacteria decreased in relative abundance with depth post-fire (Fig. 1a). Despite these trends, increased and decreased relative abundances of archaea and bacteria, respectively, reached statistical significance only at deeper depths (Pairwise *t* test, *p* < 0.001). While archaeal diversity did not differ significantly at any depth post-fire (Fig. 1b, ANOVA, *p* = 0.42), bacterial diversity was significantly lower post-fire only at deeper depths (Fig. 1c, Pairwise *t* test, *p* = 0.006).

In contrast to depth-dependent changes in alpha diversity post-fire, the composition of both archaeal and bacterial communities were significantly different between burnt and intact peat (Fig. 1d, e) (PERMANOVA, Archaea: F = 3.8, *p* = 0.003; Bacteria: F = 2.9, *p* = 0.004; Supplementary Table 1). Vertical stratification, however, remained similar within both burnt and intact peat, with communities from the surface being significantly different to those from mid (Pairwise *t* test, Archaea *p* = 0.001, Bacteria *p* = 0.005; Supplementary Table 2) and deeper (Pairwise *t* test, Archaea *p* = 0.001, Bacteria *p* = 0.03) depths. Compositional differences between mid and deeper depths, if any, could not be statistically resolved.

**Fig. 2:**
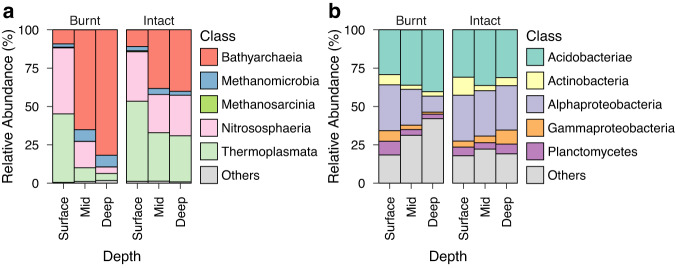
Compositional shifts across peat depth after fire. Post-fire shifts in the top five abundant classes within (**a**) archaeal and (**b**) bacterial communities. These classes were identified based on the harmonic mean of relative abundance and prevalence. Together, these classes represent approximately 68.8–81.64% of bacterial and 98.28–99.53% of archaeal communities, respectively. Mean relative abundances were computed from four independent biological replicates per group.

In light of these compositional differences across depth post-fire, we next examined shifts in the relative distributions of abundant microbial taxa in these communities. At the class level, archaeal communities were predominantly composed of ASVs from the Bathyarchaeia, Nitrososphaeria, and Thermoplasmata (Fig. 2a), while bacterial communities were mainly represented by the Acidobacteriae and Alphaproteobacteria (Fig. 2b). The relative abundances of some of these taxa, for example, the Bathyarchaeia, showed clear trends with depth post-fire, however, these differences were statistically insignificant at the surface and mid depths. Furthermore, our analysis did not identify any ASV that was significantly different post-fire at these specific depths. However, the relative abundances of several archaeal and bacterial classes and ASVs (Supplementary Tables 3 and 4) differed significantly post-fire at deeper depths. For instance, Dehalococcoidia (Wald’s test, log_2_ fold change: 6.96, *p* < 0.001), Methylomirabilia (Wald’s test, log_2_ fold change: 3.3, *p* = 0.01), and Methanomicrobia (Wald’s test, log_2_ fold change: 3.9, *p* = 0.01) significantly increased in relative abundance post-fire, whereas the Gammaproteobacteria (Wald’s test, log_2_ fold change: 1.9, *p* = 0.046) significantly decreased in relative abundance after fire. The Bathyarchaeia showed the largest post-fire increase among abundant taxa, nearly doubling in relative abundance at deeper depths (Wald’s test, log_2_ fold change: 3.4, *p* = 0.03).

### Peat fires disrupt depth-stratification of methanogens and methanotrophs

**Fig. 3:**
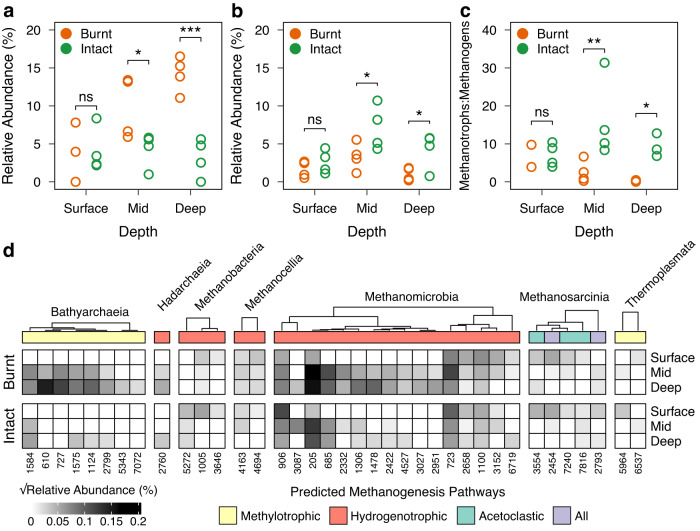
Post-fire differences in depth-stratification of functional groups involved in methane turnover. **a** Methanogens significantly increased relative to other archaea, and (**b**) methanotrophs significantly decreased relative to other bacteria at mid and deeper depths. **c** Methanotrophs-to-methanogens significantly decreased at mid and deeper depths after fire. **a–c** Represent four biological replicates per group, except for the post-fire surface in (**c**) which includes only two replicates with nonzero methanogen relative abundances. Significance was assessed using pairwise *t*-tests and is expressed as ***p < 0.001; **p < 0.01; *p < 0.05. Exact *p* values with test statistics are provided in the main text. **d** Changes in relative abundance of predicted methanogens across depth between burnt and intact sites. Each column represents an ASV, and their class labels are displayed above the tree. Mean relative abundance was computed from four biological replicates and square-root transformed for visual clarity. The colour strip at the top represents predicted methanogenesis pathways, and columns are hierarchically clustered based on Spearman-rank correlations.

To identify functional groups that potentially contribute to methane turnover, we examined the distribution of putative methanogens and methanotrophs across depths. We identified 37 archaeal taxa and 47 bacterial taxa with methanogenesis and methanotrophy potential, respectively. Methanogens and methanotrophs showed contrasting responses across depth post-fire. The relative abundance of methanogens increased (ANOVA, fire x depth, F = 7.9, *p* = 0.004) at mid (Pairwise *t* test, *p* = 0.02) and deeper (Pairwise *t* test, *p* < 0.001) depths post-fire (Fig. 3a), while conversely, methanotrophs declined in relative abundance at these depths (ANOVA, F = 6.1, *p* = 0.01) after fire (ANOVA, F = 11.6, *p* = 0.003) (Fig. 3b, mid: Pairwise *t* test, *p* = 0.01, deep: Pairwise *t* test, *p* = 0.03). These trends were further validated using metagenomic data from a subset of samples (Supplementary Fig. 2).

Methanotrophs were, on average, as abundant as methanogens in intact peat (mean ratio: 1.27 ± 0.96 s.d.) and were primarily from the Alphaproteobacteria (Supplementary Fig. 3). However, these methanotrophs decreased significantly across depths relative to methanogens after fire (Fig. 3c, mean ratio: 0.26 ± 0.28 s.d., ANOVA, F = 13.2, *p* = 0.002). On the other hand, predicted methanogens in intact peat were mainly hydrogenotrophs from the Methanomicrobia, which nearly doubled in relative abundance post-fire (Fig. 3d). Putative methanogens in burnt peat additionally comprised methylotrophic methanogens from the Bathyarchaeia, which nearly increased 10-fold in relative abundance after fire (Fig. 3d). Our metagenomic data confirmed the increase in the relative abundance of Methanomicrobia post-fire, however, only a minor fraction of retrieved *mcrA* sequences belonged to the Bathyarchaeia (Supplementary Tables 5, 6, 7).

### Selection pressure increases with depth post-fire

**Fig. 4:**
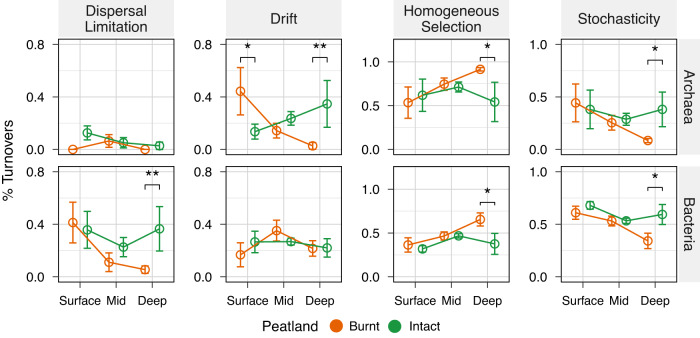
Depth-dependent shifts in the relative importance of different assembly processes after fire. Post-fire changes in dispersal limitation, drift, homogeneous selection, and stochasticity governing archaeal and bacterial community assembly across depth. Stochasticity represents the combined relative importance of drift, dispersal limitation, and homogenizing dispersal. Data (*n* = 6 comparisons among four biological replicates) are presented as mean value ± SD, with error bars representing standard deviations. Significance was assessed using one-sided bootstrap tests and is indicated as ***p < 0.001; **p < 0.01; *p < 0.05.

We next estimated assembly processes which contribute to these post-fire compositional shifts using phylogenetic bin-based null modelling. Archaeal and bacterial communities were grouped into six and sixty-four phylogenetic bins, respectively, based on phylogenetic signal (Supplementary Fig. 4). These communities were governed by homogeneous selection and ecological drift or dispersal limitation in different ways. Of these, on an average homogeneous selection contributed most to the assembly of both archaeal (mean: 67.8 ± 14.5% s.d.) and bacterial (mean: 44.1 ± 12% s.d.) communities across all samples. The relative importance of homogeneous selection increased with depth post-fire, and conversely, that of stochasticity decreased with depth for both archaeal and bacterial communities (Fig. 4, Supplementary Tables 8 and 9). Homogeneous selection was, however, significantly higher in burnt compared to intact peat only at deeper depths (Archaea, Cohen’s *d*: 2.3, *p* = 0.035; Bacteria, Cohen’s *d*: 2.8, *p* = 0.017). At this depth, homogeneous selection contributed to 91.5 ± 2% s.d. of archaeal community turnovers and 65.5 ± 7.5% s.d. of bacterial community turnovers after fire. In contrast, homogeneous selection governed only 54.8 ± 22.4% s.d. of archaeal community turnovers and 37.4 ± 12% s.d. of bacterial community turnovers in intact peat. This observation was also consistent with analysis of multivariate dispersions, which showed that communities in burnt peat were significantly more similar to each other compared to those in intact peat (PERMDISP, *p* = 0.03) at deeper depths. In contrast, stochastic processes, which decreased in relative importance post-fire, were different for archaeal and bacterial communities. Specifically, the relative importance of ecological drift and dispersal limitation in governing archaeal and bacterial community assembly, respectively, decreased with depth post-fire, with this difference becoming statistically significant at deeper depths (Fig. 4; Supplementary Tables 8 and 9).

We next performed Mantel tests to identify peat pore water parameters most associated with homogeneous selection. These tests revealed that post-fire homogeneous selection at the surface was moderated by temperature for both archaea (mean temperature, *R*: 0.86, *p* = 0.04) and bacteria (temperature variability, *R*: –0.63, *p* = 0.04). Additionally, selection on archaeal and bacterial communities was associated with pH (*R*: 0.69, *p* = 0.04) and DO variability (*R*: 0.49, *p* = 0.04), respectively. In contrast, these relationships were reversed in intact peat (Supplementary Table 10). At mid and deeper depths, post-fire homogeneous selection on bacteria was linked to mean DO (*R*: 0.86, *p* = 0.04) and temperature homogeneity (*R*: –0.5, *p* = 0.04), respectively. Conversely, selection on archaeal communities increased at deeper depths in response to greater pH variability (*R*: 0.53, *p* = 0.04).

**Fig. 5:**
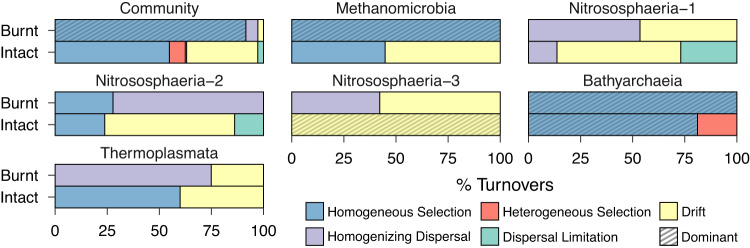
Post-fire shifts in the relative importance of assembly processes governing different archaeal groups at deeper depth. Shaded bars represent the dominant assembly process governing a particular phylogenetic bin. This was identified qualitatively as the process with the highest relative importance. Data (*n* = 6 comparisons among four biological replicates) are presented as mean values.

Finally, to further understand post-fire shifts in methanogen assembly and bins driving homogeneous selection at deeper depths, we examined within-bin process distributions and bin contributions to community assembly. This showed that Bin 5, primarily comprising the Bathyarchaeia, and Bin 1, consisting of the Methanomicrobia, both of which include methanogens, were predominantly governed by homogeneous selection after fire (Fig. 5). The Bathyarchaeia contributed most (83.3%) to archaeal community turnovers under homogeneous selection, with the Methanomicrobia being the next (7.9%) (Supplementary Table 11). On the other hand, bacterial community turnovers under homogeneous selection after fire were mainly accounted for by bins, comprised of members from the Acidobacteriae Group 13 (Bin 55: 29.9%), Desulfobacterota (Bin 15: 16.7%), and Chloroflexi (Bin 44: 6.9%), respectively (Supplementary Table 12).

## Discussion

Here, we provide evidence that post-fire changes in a tropical peatland are not only limited to hydrology, vegetation, pore water quality, and carbon dynamics but also extend to peat microbiomes and ecological processes shaping their assembly across depth. Post-fire differences in alpha diversity and relative abundances were kingdom-specific and depth-dependent, while shifts in bacterial and archaeal composition occurred across all depths (Fig. 1). Compositional changes extended to functional groups involved in methane turnover, whose relative distributions along depth were strongly altered after fire (Fig. 3). Finally, we show that assembly processes which govern compositional shifts remain strongly altered at deeper depths and are associated with post-fire environmental conditions. Collectively, our findings highlight that the effects of fires on the peat microbiome are not limited to the surface but occur at depth and are moderated by post-fire environmental conditions.

Peat depths profiled in this study capture the altered relationships between the peat surface, water table, and vegetation after fire. As previously described^9^, firstly, the peat surface post-fire was significantly lower and, therefore, much closer to the water table. Consequently, the burnt site experienced prolonged flooding during the wet season, which was further aggravated by the compacted surface and the absence of microtopography. These together contributed to lower pore water DO and EC, with the latter also serving as a proxy for nutrient leaching. Secondly, the vegetation canopy was replaced by sparse, shallow-rooted graminoid cover, which in turn contributed to higher peat surface temperatures, pore water temperatures, and higher methane emissions due to direct gas transport through the plant aerenchyma^9,29^. Lower DO was further attributed to limited diffusion at higher temperatures and could also be exacerbated at depth due to the absence of root oxygenation^30^. Apart from these indirect effects, only changes in pH were directly attributed to fire, likely due to fire-derived modifications of organic acids^31^. In line with our previous measurements, pore water pH and temperature remained elevated post-fire. Conversely, differences in DO and EC were minimal, consistent with their patterns during the dry season^9^.

The extent to which historical and contemporary post-fire environmental conditions influence microbiome shifts can be inferred from the ecological processes governing their assembly^32^. Selection implies that these shifts are coupled to environmental conditions, whereas stochasticity indicates that they are linked to random events such as dispersal and drift^33,34^. The balance between selection and dispersal moderates alpha diversity across different ecosystems^35,36^. In this study, alpha diversity did not differ at the surface and mid depths three years since fire, which can likely be explained by hydrologic dispersal through time as both sites lie within the same peat dome^9^. This is corroborated by dispersal limitation and selection pressures being comparable across both sites at these depths (Fig. 4). In contrast to alpha diversity, beta diversity differed at these depths and was influenced by both selection and stochasticity. Homogeneous selection on archaeal and bacterial communities was moderated by pore water temperature, DO, and pH to different extents in a depth-dependent manner, in line with previous studies that identified them as significant drivers of peat microbiome composition^37–39^. Unlike homogeneous selection, the influence of drift and dispersal was domain-specific and can likely be explained by lower archaeal and higher bacterial relative abundances within the community, respectively. Ecological drift, which leads to turnover due to random extinction events, becomes prominent at lower species frequencies, while conversely, dispersal increases with increased species relative abundances^33^. Bacterial turnovers governed by dispersal limitation at these depths may arise due to seasonal differences in hydrological connectivity^9,37^. Despite observing turnover at these depths, our analysis did not detect any differentially abundant taxa. This discrepancy likely results either from our use of conservative shrinkage estimates^40^ or from our limited sample size, which may preclude the detection of smaller effect sizes^41^, given the variability at these depths compared to deeper depths.

In contrast to the surface and mid depths, the decreased bacterial diversity and the shift in the relative abundances of bacteria and archaea at deeper depths can be attributed to strong selective pressures at this depth. Specifically, decreased variability in pore water temperatures post-fire may homogenise bacterial communities, similar to effects observed in soil warming experiments^26,42^. However, consistent with previous studies in post-fire mineral soils^43,44^, archaeal diversity did not differ after fire despite the presence of similar selective pressures, such as variability in pH, on the community. This, together with their increased relative abundance, suggests that diverse archaeal groups are able to colonize and persist in the highly selective post-fire conditions at deeper depths compared to bacteria. This interpretation is consistent with their capacity to tolerate stressful conditions across different ecosystems^45^. While selective pressures did not influence archaeal diversity, they strongly contributed to both archaeal and bacterial community turnovers at deeper depths. Interestingly, the positive association between selection on archaeal communities and pH heterogeneity post-fire suggests that selection mechanisms may either involve pressures imposed by unmeasured environmental variables or extend beyond environmental homogeneity and/or extreme conditions. For instance, in a soil warming experiment, homogeneous selection on bacterial communities was linked to both temperature homogeneity and plant biomass heterogeneity^26^. In contrast, a possible scenario in which homogeneous selection might occur in a heterogeneous environment is when selection favours generalists^46^. These possibilities merit future investigations that couple comprehensive environmental characterization with trait-based approaches. Furthermore, selection operates on microbial traits and may manifest as trait differences among abundant microbial taxa. For instance, we observed an increase in the relative abundance of the Bathyarchaeia, which are known to be abundant in anoxic environments with refractory carbon^47^. Conversely, the post-fire increase in pH likely contributed to the decrease in the relative abundance of pH-sensitive groups such as the Thermoplasmata^48,49^.

The relative importance of different assembly processes may also offer insights into microbiome successional stage and, hence, recovery after the fire disturbance. Deterministic processes predominantly govern microbiome assembly during mid-successional stages due to the highly restrictive post-fire environmental conditions during this period^19,22^. On the other hand, stochastic processes have been proposed to progressively increase during the later stages of recovery^22^. Given this, our data suggests successional stages and, hence, microbiome recovery after fire may vary with depth, with microbiomes at deeper depths recovering more slowly compared to those at the surface and mid depths. Moreover, taxonomic shifts along depth appear to reflect a successional gradient, further reinforcing our hypothesis that microbiome succession and, hence, recovery varies with depth. For example, Proteobacteria, which are typically more abundant in late-successional landscapes^19,50^, showed a decreasing trend with depth post-fire. Nonetheless, these shifts require careful examination at multiple taxonomic levels, given that groups such as Actinobacteria and Acidobacteriae, which are typically more abundant in early to mid-successional communities^13^, showed an increasing trend with depth.

Compositional shifts along depth were not restricted to taxonomic groups but also extended to functional groups involved in methane turnover. Lower DO, coupled with increased pH and temperature post-fire, as outlined earlier, may explain the observed shifts in methanotrophs-to-methanogens with depth. This interpretation aligns with both previous reports^51^ and our experiments, which linked increased methane emissions to higher temperatures under anoxic conditions^28^. Further, post-fire environmental conditions enriched for specific methanogens, particularly at mid and deeper depths, as corroborated by their assembly being largely governed by homogeneous selection. The enrichment of putative methylotrophic methanogens can be attributed to the hydrologic transport of recently-fixed, reactive photosynthates from the surface to deeper depths. This interpretation is corroborated by radiocarbon signatures and lower dissolved organic carbon levels in burnt peat from our previous study^9^ and further supported by prior research^52–55^. On the other hand, the enrichment of hydrogenotrophic methanogens may be fuelled by substrates derived from the fermentation of labile root exudates. This view is supported by our earlier study, which showed that an increase in methane emissions from burnt peat could be triggered by an artificial combination of plant sugars and fermentative end products such as acetate and formate^28^. Finally, the co-existence of abundant methanogens could be explained by niche differentiation through the use of competitive and non-competitive methanogenic substrates^56–59^.

Unexpectedly, predicted methanogens from the class Bathyarchaeia showed the greatest enrichment with depth post-fire and reached similar levels in relative abundance as that of the Methanomicrobia. However, we did observe a discrepancy in the relative abundance of Bathyarchaeial methanogens between our predicted and sequenced metagenomes. Firstly, this may reflect the reduced sensitivity of current *mcrA* gene HMM models to detect highly divergent *mcrA* sequences from the Bathyarchaeia, as existing models are constructed largely from sequences from classical methanogens. Secondly, this discrepancy may arise due to overprediction by PICRUST2 due to the limited number of reference genomes in its database^60^. Nonetheless, the detection of *mcrA* genes belonging to the Bathyarchaeia in our sequenced metagenomes confirms their presence in burnt peat and suggests their potential role in methane emissions post-fire. However, these findings need further validation as methanogenic markers have only been detected in a limited number of species from this lineage and the current lack of definitive physiological evidence of their capacity to produce methane^61^.

An important limitation of our study is that it represents a snapshot in time despite being informed by long-term monitoring of environmental parameters and specific ecosystem functions. Microbiome functioning likely varies with seasonal changes in precipitation, as evidenced by temporal variability in methane emissions^9^. This underscores the need for long-term or space-for-time substitution studies, which capture successional changes in microbiome composition and functioning after fire. With respect to the findings presented here, three important caveats are sample size, abundance measurements, and activity measurements. Firstly, our limited sample size may have constrained our capacity to obtain precise estimates of the environmental factors driving assembly processes. Secondly, we acknowledge that the changes in relative abundance may not necessarily reflect changes in absolute abundance. Finally, functional potential does not necessarily correlate with activity and thus requires additional validation using metatranscriptomics or metaproteomics.

Despite these limitations, our findings underscore essential ecological principles that can inform efforts to predict emissions and guide the restoration of fire-affected tropical peatlands. Firstly, ecosystem functions in extensive fire-affected peatlands, like methane turnover, can potentially be predicted solely based on environmental conditions, given the heightened homogeneous selection with depth. Secondly, efforts to mitigate methane emissions could prioritize the restoration of woody aboveground vegetation to enhance shading and promote oxygenation at depth. Finally, given the strong post-fire selective pressures, plant restoration strategies such as assisted natural regeneration could include compatible post-fire adapted microorganisms in rhizosphere inoculants to improve plant establishment. Given the vulnerability of a significant portion of tropical peatlands to fires and climate change^5^, it is crucial to harness predictive ecological principles to improve emissions forecasting and inform post-fire recovery strategies.

## Methods

### Site description, peat sampling, and pore water analysis

As previously described^9^, our study site is a tropical peatland located in the Belait district, Brunei Darussalam. This region experiences an equatorial-humid climate with a mean monthly temperature of 27.2 ± 0.4 °C and mean annual precipitation of 3000 mm based on records between 1955 and 2018. The wet period occurs between October to January and May to June, while the dry period occurs in the intervening months. Fires typically occur during the dry season with seven fire events occurring at our burnt site between 1998-2016. The last fire event was recorded in March 2016. Water table levels fluctuated by as much as 30 cm above and below the mean peat surface during the wet and dry seasons, respectively. Water table levels were on an average 5–10 cm higher in the burnt transect during the wet season, however, the converse was true during the dry season. Post-fire vegetation mainly consisted of ferns and flood tolerant, shallow rooted graminoids such as sedges (*Scleria sumatrensis*). In contrast, the undisturbed forest site consisted of deep rooted, woody tree species such as *Shorea albida* mixed with *Pandanus andersonii*, and *Pandanus helicopus*.

Peat samples for this study were collected from three different depths (Surface: 0–5 cm, Mid: 35–40 cm, and Deep: 95–100 cm; *n* = 4 per depth) in August 2018 from a transect each at the burnt and intact site. Transect and plot establishment have been previously described in detail^9^. Our time of sampling coincided with the dry season. Samples were collected using a Russian auger and transferred into 50 ml tubes. Tubes were immediately stored on ice on site and transported to the laboratory within 24 h where they were transferred to deep freezers until processing. Pore water quality was assessed on site using a multi parameter probe (YSI556 Multiprobe, YSI Life Sciences, USA). Parameters recorded included dissolved oxygen (DO), electrical conductivity (EC), total dissolved solids (TDS), salinity, water temperature, and pH. As TDS and salinity are derived from EC measurements and hence collinear, we only considered EC for our statistical analysis.

### DNA extraction and 16S rRNA marker gene sequencing

Genomic DNA was extracted from all peat samples using the ZymoBIOMICS DNA miniprep kit (Zymo Research, Irvine, CA, USA). Sequence libraries were prepared by amplifying the V4-V5 regions of the 16S rRNA gene from the extracted DNA using the 515F (5ʹ-GTG YCA GCM GCC GCG GTAA-3ʹ) and 926R (5ʹ-CCG YCA ATT YMT TTR AGT TT-3ʹ) primers tagged with Illumina overhang adapters. Libraries were sequenced on the Illumina MiSeq (Illumina, San Diego, CA, USA) with 2×300 bp chemistry at SCELSE (https://www.scelse.sg), Nanyang Technological University, Singapore. We generated a total of 8.7 M paired-end reads, with each sample containing on an average 80,105 reads.

### Sequence analyses

Raw sequence data was processed using Cutadapt v3.4^62^ to remove Illumina adapters and phiX sequences. Adapter-free reads were trimmed (forward: 230 bp, reverse: 180 bp) and filtered to retain only those with a maximum error rate (maxEE) ≤ 3. Overall, 6.1 M reads (approx. 70%) were retained post-filtering. Amplicon sequence variants (ASVs) were identified from filtered reads using the DADA2^63^ pipeline. Chimera-free ASVs were filtered to retain only those with length 364 ± 10 bp. ASV counts were estimated by mapping filtered reads to length-filtered, chimera-free ASVs. Taxonomic labels were inferred using the SILVA database v138.1^64^. A phylogenetic tree was built using FastTree2^65^, with ASV sequences aligned using the DECIPHER^66^ R package.

### Metagenome sequencing

Shotgun metagenomes were generated from 13 samples with sufficient genomic DNA remaining after amplicon sequencing. Samples were sequenced on the Illumina HiSeqX (Illumina, San Diego, CA, USA) with 2×150 bp chemistry at SCELSE (https://www.scelse.sg), Nanyang Technological University, Singapore. We generated a total of 0.23 Tbp of raw sequence data. Raw sequence reads were processed using Cutadapt v3.4^62^ with parameters: --error-rate 0.2, --minimum-length 75, --no-indels, to remove Illumina adapters. Low-quality regions from adapter-free reads were then trimmed using bbmap v38.96 (https://sourceforge.net/projects/bbmap/) with parameters: trimq = 20, qtrim = rl, minlen = 75.

### Microbiome diversity and compositional analysis

Compositional differences between fire-affected and intact peat samples, and across depth were analysed using R v4.2.2^67^ and PRIMER-E v7 (https://www.primer-e.com/). Bray-Curtis dissimilarity between samples were computed using Hellinger-transformed ASV counts. Between-sample variability due to fire and depth were quantified using permutational analysis of variance (PERMANOVA) implemented in PRIMER-E v7, and visualised using canonical analysis of principle coordinates (CAP) with the phyloseq v1.42.0^68^ and vegan v2.6.4^69^ R packages.

Datasets for all analysis were cleaned and wrangled using the tidyverse v1.3.2^70^ R package. Archaea-to-bacteria ratio was computed using total sum scaled (TSS) transformed ASV counts. Within-sample diversity was estimated using the Shannon diversity metric with raw counts. Differences in archaea-to-bacteria ratio, diversity, and methanogen/methanotroph relative abundances were analysed using ANOVA. Post-hoc tests were conducted using the emmeans R package; *p* values were adjusted using the mvt method. The harmonic mean of the global relative abundance and global prevalence (occurrence frequency across samples) of each ASV was used to identify taxa that were consistently dominant in burnt and intact communities. The magnitude of change in an ASVs relative abundance with fire and depth was quantified using differential abundance analysis implemented in the DESeq2 v1.38.3^41^ R package. Log_2_ fold changes were shrunk using the apeglm v1.20.0^40^ R package for ASVs, with either low information across samples or high dispersion; *p* values, were adjusted for multiple testing. Significantly different taxa were identified as those with an adjusted *p* value < 0.05.

### Functional groups involved in methane turnover

KEGG profiles were predicted for all ASVs using PICRUST2. Predictions were only retained for those ASVs with a Nearest Sequenced Taxon Index (NSTI) value ≤ 2 to eliminate those that could not be placed on the reference tree with adequate confidence^71^. From these profiles, we identified methanogens and methanotrophs as those ASVs having KEGG modules associated with these pathways (completeness ≥75%). Module completeness was computed using enrichM v0.6.5 (https://github.com/geronimp/enrichM). Predicted modules were manually curated by cross-referencing them with literature, when available^72^.

Methanogens and methanotrophs were profiled from quality-trimmed metagenomes using a read-based approach with graftM^73^. Methyl-coenzyme M reductase A (*mcrA*) and the particulate methane monooxygenase (*pmoA*) genes were used as markers for methanogens and methanotrophs, respectively. Counts associated with each marker were normalized against the counts of the universal single-copy gene ribosomal protein S10 (*rpsJ*) to approximate relative abundances in each sample. Correlations between the relative abundances of these functional groups from predicted and sequenced metagenomes were evaluated using the Spearman’s rank correlation coefficient.

### Assembly processes

Clade- and community-level assembly processes were estimated using a phylogenetic bin-based null modelling approach, implemented in the iCAMP v1.5.12^26^ R package. Significant phylogenetic signal is a pre-requisite for estimating assembly processes. Therefore, phylogenetic signal was estimated by regressing between-ASV phylogenetic distances against between-ASV niche differences in a pairwise manner and tested for statistical significance using a Mantel test (Mantel *R* ≥ 0.1, *p* < 0.05). The distance within which phylogenetic signal was consistently observed, was used as the phylogenetic distance threshold for demarcating phylogenetic bins. Post-fire changes in the assembly of entire communities and individual clades were tested by computing standardised effect sizes (Cohen’s *d*) and tested using a one-sided bootstrap test (1000 times). Cohen’s *d* was computed as the difference in means between the burnt and intact samples divided by the combined standard deviation. Computed effect sizes were classified as large ( | *d*| > 0.8), medium (0.5 < |*d*| ≤ 0.8), small (0.2 < |*d*| ≤ 0.5), or negligible ( |*d*| ≤ 0.2). The process with highest relative importance was considered the dominant process governing a pairwise turnover^26^.

### Relationships between assembly processes and physicochemical conditions

Correlations between the relative importance of assembly processes and environmental variables were tested using Mantel tests implemented in the iCAMP^26^ R package. We computed associations between pairwise differences in the relative importance of each assembly process with either the variation (absolute difference) or mean of every environmental variable. All environmental variables except pH were log-transformed in order to explore log-linear relationships.

### Reporting summary

Further information on research design is available in the Nature Research Reporting Summary linked to this article.

### Supplementary information

Supplementary Information

Supplementary Tables

Reporting Summary

## Data Availability

Raw sequence data is available on the NCBI Sequence Read Archive (SRA) under BioProject PRJNA772087.

## Code availability

R codes used to analyse the data are available on GitHub (https://github.com/adityabandla/2023-peat-microbiome-post-fire).
