# Supplementary material for: Elevated methane flux in a tropical peatland post-fire is linked to depth-dependent changes in peat microbiome assembly
Source: NPJ Biofilms Microbiomes. 2024 Jan 23;10:8. doi: 10.1038/s41522-024-00478-9 (PMC10803758; doi:10.1038/s41522-024-00478-9)
Supplement: Supplementary file 1 — Supplementary Information [file 41522_2024_478_MOESM1_ESM.pdf]

Supplementary information for

Elevated methane flux in a tropical peatland post-fire is linked to depth-dependent changes in microbiome assembly

Aditya Bandla, Hasan Akhtar, Massimo Lupascu, Rahayu Sukmaria Sukri, Sanjay Swarup

Corresponding author: Sanjay Swarup (sanjay@nus.edu.sg)

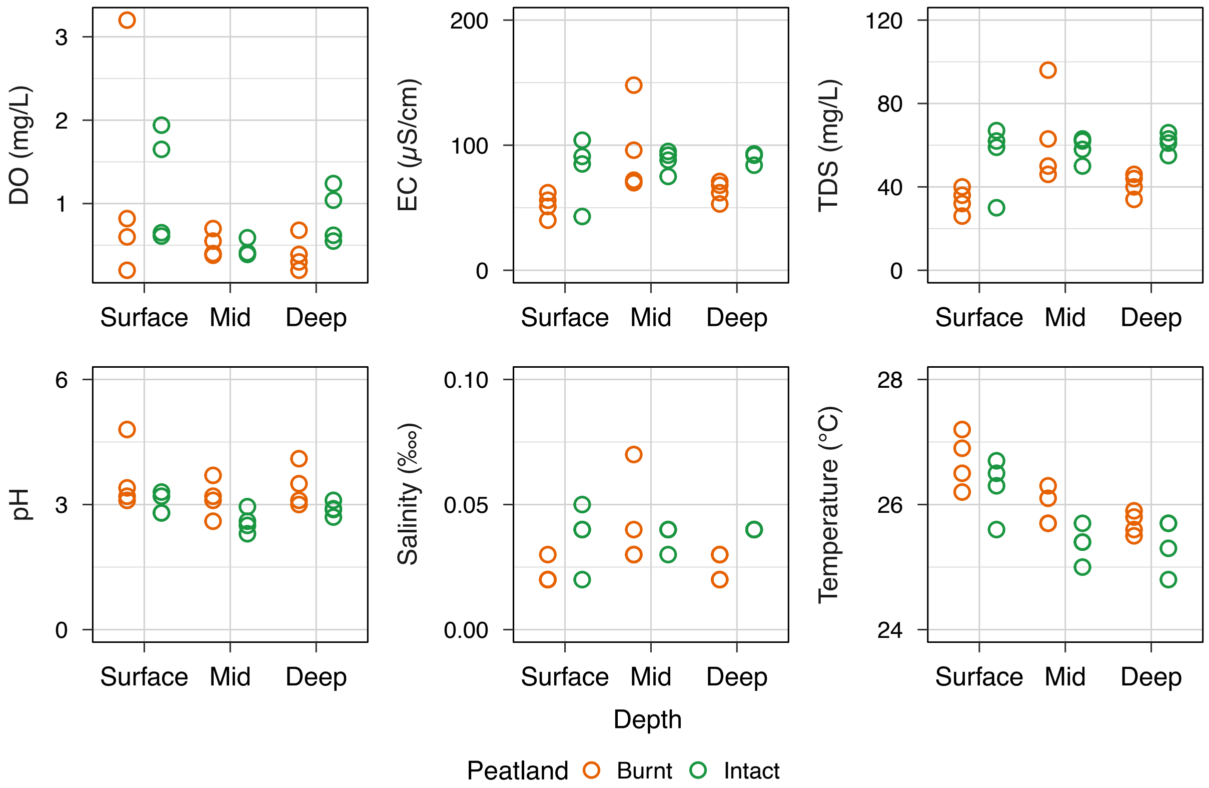

Supplementary Figure 1: Peat pore water quality remains strongly altered three-years post-fire.

Supplementary Table 1: PERMANOVA summary statistics using Type III Sums of Squares. Fire and depth were considered as fixed factors. P-values were estimated based on 999 permutations.

	Factor	Pseudo-F	P-value	R^2
Archaea	Fire	3.79	0.003	18.43
	Depth	5.34	0.001	28.15
	Fire:Depth	1.27	0.247	9.98
Bacteria	Fire	2.88	0.004	16.38
	Depth	2.86	0.006	19.92
	Fire:Depth	1.32	0.180	11.62

Supplementary Table 2: PERMANOVA pairwise comparisons for depth.

	Depth	t-value	P-value
Archaea	Surface v Mid	2.51	0.001
	Surface v Deep	2.64	0.001
	Mid v Deep	1.41	0.068
Bacteria	Surface v Mid	2.05	0.005
	Surface v Deep	1.69	0.031
	Mid v Deep	1.23	0.159

Supplementary Table 3: Significantly enriched or depleted bacterial and archaeal classes in burnt peat at deeper depth. Table is presented in a separate spreadsheet.

Supplementary Table 4: Significantly enriched or depleted bacterial and archaeal ASVs in burnt peat at deeper depth. Table is presented in a separate spreadsheet.

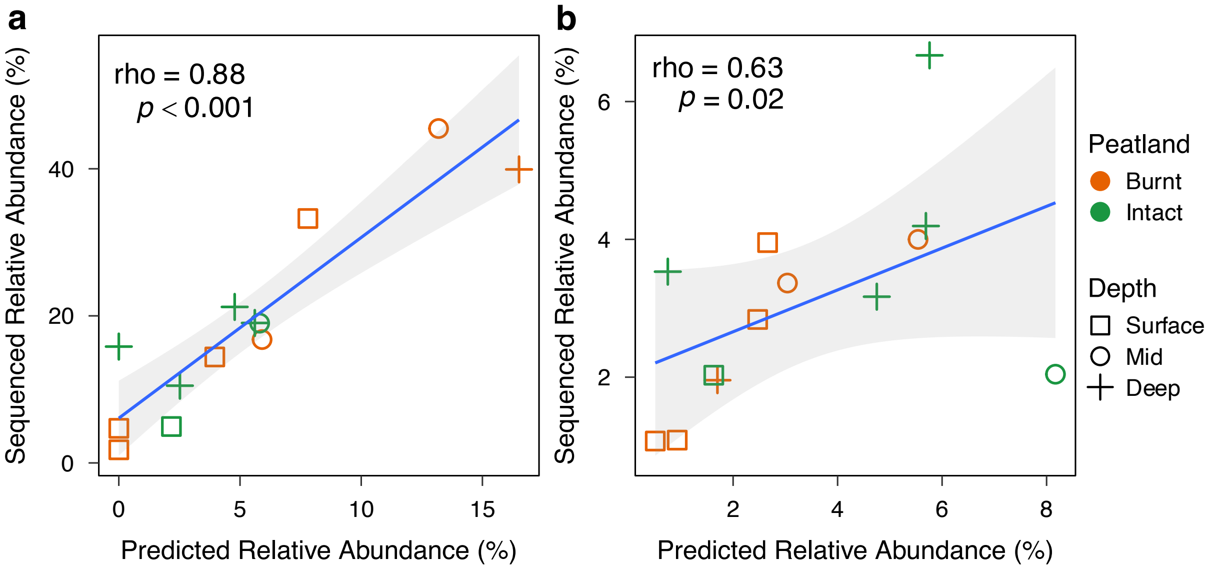

Supplementary Figure 2: Correlations between relative abundances of functional groups in predicted and sequenced metagenomes. ASVs constituting **a** methanogens and **b** methanotrophs were identified based on predicted KEGG profiles. Relative abundances represent the combined abundances of taxa within each group. Methyl-coenzyme M reductase A and Particulate methane monooxygenase A genes served as markers for estimating the relative abundances of methanogens and methanotrophs from sequenced metagenomes, respectively. Marker gene abundances were normalized using the single-copy ribosomal protein S10 marker

gene. Spearman rank correlations were employed to calculate the correlations between predicted and sequenced metagenomes.

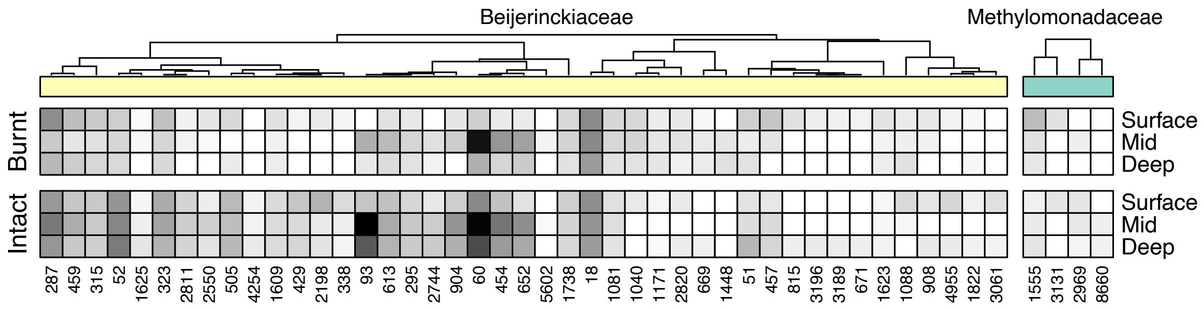

Supplementary Figure 3: Changes in relative abundance of predicted methanotrophs across depth between burnt and intact sites. Each column corresponds to an ASV, with their respective families indicated by a color strip at the top, and their names displayed above the tree. Mean relative abundance was computed from four biological replicates and square-root transformed for visual clarity. Columns are hierarchically clustered based on Spearman-rank correlations.

Supplementary Table 5: Summary of sequenced shotgun metagenomes. Table is presented in a separate spreadsheet.

Supplementary Table 6: Relative abundance of Metagenome Sequence Variants (MSV) of the Methyl-coenzyme M reductase A gene identified from sequenced metagenomes. Abundance was normalised using the single-copy ribosomal protein S10 gene corresponding to the archaea. Table is presented in a separate spreadsheet.

Supplementary Table 7: Relative abundance of Metagenome Sequence Variants (MSV) of the Particulate methane monooxygenase A gene identified from sequenced metagenomes. Abundance was normalised using the single-copy ribosomal protein S10 gene corresponding to the bacteria. Table is presented in a separate spreadsheet.

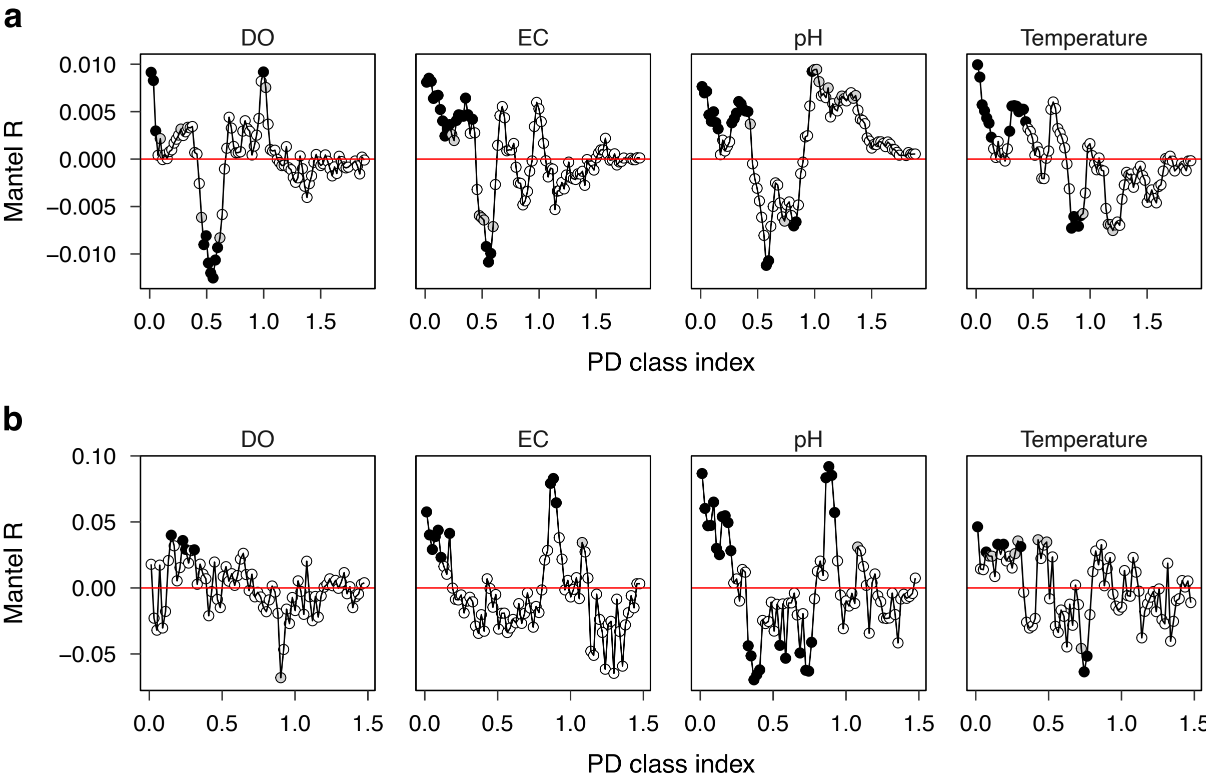

Supplementary Figure 4. Significant phylogenetic signal was detected across short phylogenetic distances in both **a** archaeal and **b** bacterial communities. Mantel correlograms were constructed by regressing niche differences between ASVs against phylogenetic distances. A positive association indicates that ecological niche distance increases with phylogenetic distance within the specified distance class. Filled symbols (black: $p<0.05$; grey: $p<0.1$) represent significant associations within a particular distance class, while unfilled symbols indicate non-significant associations.

Supplementary Table 8: Relative importance of different ecological processes structuring archaeal communities in burnt and intact peat at deeper depth. Values for burnt and intact peat show observed values (with standard deviations). Statistical significance was estimated using a one-sided bootstrap test.

Process	Burnt	Intact	d	Effect size	P
Homogeneous selection	91.54 (1.96)	54.83 (22.45)	2.34	Large	0.035
Heterogeneous selection	0.00 (0.00)	7.63 (7.93)	-1.36		0.38
Ecological drift	2.70 (2.58)	34.10 (17.79)	-2.52	Large	0.008
Homogenizing Dispersal	5.76 (2.08)	0.64 (0.66)	3.31	Large	0.05
Dispersal limitation	0.00 (0.00)	2.80 (2.85)	-1.38		0.38

Supplementary Table 9: Relative importance of different ecological processes structuring bacterial communities in burnt and intact peat at deeper depth. Values for burnt and intact peat show observed values (with standard deviations). Statistical significance was estimated using a one-sided bootstrap test.

Process	Burnt	Intact	d	Effect size	P
Homogeneous selection	65.30 (7.47)	37.35 (11.96)	2.80	Large	0.02
Heterogeneous selection	0.38 (0.25)	3.07 (2.68)	-1.45		0.32
Ecological drift	21.71 (5.89)	21.85 (7.04)	-0.06		0.50
Homogenizing Dispersal	7.11 (1.99)	0.81 (0.80)	4.14	Large	0.003
Dispersal limitation	5.50 (2.88)	36.92 (16.91)	-2.57	Large	0.007

Supplementary Table 10: Pore water parameters significantly associated (p=0.04) with homogeneous selection across burnt and intact peat. Correlations and their significance were assessed using Mantel tests.

Variable	R	Type	Depth	Kingdom
Mean temperature	0.86	Burnt	Surface	Archaea
Δ pH	0.69	Burnt	Surface	Archaea
Δ pH	-0.38	Intact	Surface	Archaea
Δ EC	-0.72	Intact	Surface	Archaea
Δ pH	0.83	Intact	Mid	Archaea
Δ pH	0.53	Burnt	Deep	Archaea
Δ Temperature	-0.63	Burnt	Surface	Bacteria
Δ DO	0.49	Burnt	Surface	Bacteria
Δ Temperature	0.65	Intact	Surface	Bacteria
Δ EC	-0.64	Intact	Surface	Bacteria
Δ DO	-0.81	Intact	Surface	Bacteria
Mean DO	0.86	Burnt	Mid	Bacteria
Δ Temperature	-0.50	Burnt	Deep	Bacteria

Supplementary Table 11: Relative contributions of different archaeal phylogenetic bins to community-level assembly processes. Relative abundances provided in the look up table correspond to global mean relative abundances of the respective phylogenetic bin. Table is presented in a separate spreadsheet.

Supplementary Table 12: Relative contributions of different bacterial phylogenetic bins to community-level assembly processes. Relative abundances provided in the look up table correspond to global mean relative abundances of the respective phylogenetic bin. Table is presented in a separate spreadsheet.
