# Supplementary material for: Elevated methane flux in a tropical peatland post-fire is linked to depth-dependent changes in peat microbiome assembly
Source: NPJ Biofilms Microbiomes. 2024 Jan 23;10:8. doi: 10.1038/s41522-024-00478-9 (PMC10803758; doi:10.1038/s41522-024-00478-9)
Supplement: Supplementary file 3 — Reporting Summary [file 41522_2024_478_MOESM3_ESM.pdf]

nature portfolio

Corresponding author(s): Sanjay Swarup

Last updated by author(s): Jan 4, 2024

Reporting Summary

Nature Portfolio wishes to improve the reproducibility of the work that we publish. This form provides structure for consistency and transparency in reporting. For further information on Nature Portfolio policies, see our Editorial Policies and the Editorial Policy Checklist.

Statistics

For all statistical analyses, confirm that the following items are present in the figure legend, table legend, main text, or Methods section.

n/a	Confirmed	
☐	☒	The exact sample size (*n*) for each experimental group/condition, given as a discrete number and unit of measurement
☐	☒	A statement on whether measurements were taken from distinct samples or whether the same sample was measured repeatedly
☐	☒	The statistical test(s) used AND whether they are one- or two-sided<br>*Only common tests should be described solely by name; describe more complex techniques in the Methods section.*
☐	☒	A description of all covariates tested
☐	☒	A description of any assumptions or corrections, such as tests of normality and adjustment for multiple comparisons
☐	☒	A full description of the statistical parameters including central tendency (e.g. means) or other basic estimates (e.g. regression coefficient) AND variation (e.g. standard deviation) or associated estimates of uncertainty (e.g. confidence intervals)
☐	☒	For null hypothesis testing, the test statistic (e.g. *F*, *t*, *r*) with confidence intervals, effect sizes, degrees of freedom and *P* value noted<br>*Give P values as exact values whenever suitable.*
☒	☐	For Bayesian analysis, information on the choice of priors and Markov chain Monte Carlo settings
☒	☐	For hierarchical and complex designs, identification of the appropriate level for tests and full reporting of outcomes
☐	☒	Estimates of effect sizes (e.g. Cohen's *d*, Pearson's *r*), indicating how they were calculated

Our web collection on statistics for biologists contains articles on many of the points above.

Software and code

Policy information about availability of computer code

Data collection	No software was used for data collection.
Data analysis	R v4.2.2, Cutadapt v3.4, DADA2 v1.28.0, FastTree v2.1.11, DECIPHER v2.26.0, PRIMER-E v7, phyloseq v1.42.0, vegan v2.6.4, tidyverse v1.3.2, DESeq2 v1.38.3, apeglm v1.20.0, PICRUST2, enrichM v0.6.5, graftM v0.14.0, iCAMP v1.5.12, emmeans v1.8.7. All R scripts used in this manuscript are available on GitHub (https://github.com/adityabandla/2023-peat-microbiome-post-fire)

For manuscripts utilizing custom algorithms or software that are central to the research but not yet described in published literature, software must be made available to editors and reviewers. We strongly encourage code deposition in a community repository (e.g. GitHub). See the Nature Portfolio guidelines for submitting code & software for further information.

Data

Policy information about availability of data

All manuscripts must include a data availability statement. This statement should provide the following information, where applicable:

- Accession codes, unique identifiers, or web links for publicly available datasets
- A description of any restrictions on data availability
- For clinical datasets or third party data, please ensure that the statement adheres to our policy

Raw sequence data is available on the NCBI Sequence Read Archive (SRA) under BioProject PRJNA772087.

Research involving human participants, their data, or biological material

Policy information about studies with human participants or human data. See also policy information about sex, gender (identity/presentation), and sexual orientation and race, ethnicity and racism.

Reporting on sex and gender	N/A to this study.
Reporting on race, ethnicity, or other socially relevant groupings	N/A to this study.
Population characteristics	N/A to this study.
Recruitment	N/A to this study.
Ethics oversight	N/A to this study.

Note that full information on the approval of the study protocol must also be provided in the manuscript.

Field-specific reporting

Please select the one below that is the best fit for your research. If you are not sure, read the appropriate sections before making your selection.

☐ Life sciences ☐ Behavioural & social sciences ☒ Ecological, evolutionary & environmental sciences

For a reference copy of the document with all sections, see nature.com/documents/nr-reporting-summary-flat.pdf

Ecological, evolutionary & environmental sciences study design

All studies must disclose on these points even when the disclosure is negative.

Study description	In this study, we investigated post-fire shifts in microbiome composition across peat depth in a tropical peatland. Peat samples were collected from four plots located along a transect within the burnt and intact sites. Plots were on an average 100-150 m apart and therefore samples collected from each plot were considered as biological replicates. In each plot, peat samples were collected from three distinct depths: Surface (0-5 cm), Mid (35-40 cm), and Deep (95-100 cm).
Research sample	Samples analysed in this study consist of peat samples collected from both an intact and burnt site in a tropical peatland. A total of 24 peat samples were collected, processed, and analysed.
Sampling strategy	Since specific differences in microbiome composition between intact and burnt sites were not known prior to sampling, we could not estimate the required sample size using statistical power analysis. Nevertheless, we expected large differences given the major shifts in the peat surface, water table, pore water quality, and aboveground vegetation post-fire reported in our previous study (Lupascu et al. 2020, https://doi.org/10.1111/gcb.15195). The sample sizes are considered sufficient as we were able to detect effects, and their significance is supported by relevant p-values.
Data collection	H.A. measured pore water quality on-site using a multi-parameter probe (YSI 556 MPS) and collected peat samples using a Russian auger with assistance from field personnel. H.A. extracted DNA, and prepared 16S rRNA gene amplicon libraries for sequencing. Libraries were sequenced on an Illumina MiSeq (Illumina, San Diego, CA, USA) at SCELSE, Nanyang Technological University, Singapore (https://www.scelse.sg).
Timing and spatial scale	Peat samples were collected in August 2018, during the dry season.
Data exclusions	No data were excluded from any of the reported analyses.
Reproducibility	We did not attempt to replicate the reported findings, since our study relies on field observations. Nevertheless, we have made the data and code used to generate our findings available to facilitate replication by other researchers.
Randomization	This study did not involve allocation of samples to experimental groups. Rather, sample categories correspond to their natural environmental state. All samples were randomized prior to DNA extraction, library preparation, and sequencing.
Blinding	Blinding was not considered for this study as the work presented here is based on field observations.

Did the study involve field work? ☒ Yes ☐ No

Field work, collection and transport

Field conditions	The study site is situated within a region which experiences an equatorial-humid climate with a mean monthly temperature of 27.2 ± 0.4°C and mean annual precipitation of 3, 000 mm based on records between 1955-2018. The wet period occurs between October to January and May to June, while the dry period occurs in the intervening months. Fires typically occur during the dry season with seven fire events recorded at our burnt site between 1998-2016. The last fire event was recorded in March 2016. Water table levels fluctuated by as much as 30 cm above and below the mean peat surface during the wet and dry seasons, respectively. Water table levels were on an average 5-10 cm higher in the burnt transect during the wet season, however, the converse was true during the dry season. Post-fire vegetation mainly consisted of ferns, and flood tolerant, shallow rooted graminoids such as sedges, in contrast to deep rooted, woody tree species such as Shorea albida mixed with Pandanus andersonii and Pandanus helicopus in the undisturbed forest site.
Location	As previously described in Lupascu et al. 2020 (https://doi.org/10.1111/gcb.15195), our study site is a peatland located in the Belait district, Brunei Darussalam. The burnt (4°28′40″N, 114°18′19″E) and intact (4°27′32″N, 114°18′57″E) sites are situated on the same peat dome.
Access & import/export	We obtained entry and sampling permits (Ref:[209]/JPH/UND/17 PT.1) from the Brunei Forestry Department, export permits from the Biodiversity Research and Innovation Centre, and phytosanitary certificates from the Biosecurity Division.
Disturbance	Pore water quality was measured first using the multiparameter probe, prior to peat sample collection. In our previous study (Lupascu et al. 2020, https://doi.org/10.1111/gcb.15195), only one core was collected from each plot in January 2018. For the current study, peat cores were collected seven months later, and the sampling location within the plot was carefully chosen to be sufficiently distant from the previous core. Therefore, we anticipate minimal disturbances to the collected samples.

Reporting for specific materials, systems and methods

We require information from authors about some types of materials, experimental systems and methods used in many studies. Here, indicate whether each material, system or method listed is relevant to your study. If you are not sure if a list item applies to your research, read the appropriate section before selecting a response.

Materials & experimental systems

n/a	Involved in the study
☒	☐ Antibodies
☒	☐ Eukaryotic cell lines
☒	☐ Palaeontology and archaeology
☒	☐ Animals and other organisms
☒	☐ Clinical data
☒	☐ Dual use research of concern
☒	☐ Plants

Methods

n/a	Involved in the study
☒	☐ ChIP-seq
☒	☐ Flow cytometry
☒	☐ MRI-based neuroimaging

Plants

Seed stocks	N/A to this study.
Novel plant genotypes	N/A to this study.
Authentication	N/A to this study.
